# Hospital organizational change: The importance of teamwork culture, communication, and change readiness

**DOI:** 10.3389/fpubh.2023.1089252

**Published:** 2023-02-09

**Authors:** Louise A. Ellis, Yvonne Tran, Chiara Pomare, Janet C. Long, Kate Churruca, Maree Saba, Jeffrey Braithwaite

**Affiliations:** Faculty of Medicine, Health, and Human Sciences, Centre for Healthcare Resilience and Implementation Science, Australian Institute of Health Innovation, Macquarie University, Macquarie, NSW, Australia

**Keywords:** organizational change, organizational culture, workplace culture, communication, change management, change readiness

## Abstract

**Background:**

Hospital organizational change can be a challenging time, especially when staff do not feel informed and ready for the change to come. A supportive workplace culture can mitigate the negative effects allowing for a smooth transition during hospital organizational change. In this paper, we test an exploratory path model by which teamwork culture influences staff attitudes in feeling informed and ready for change, and which are ultimately related to reduced staff burnout. We also examined different types of change communication, identifying the channels that were perceived as most useful for communicating organizational change.

**Methods:**

In 2019, a cross-sectional online and paper-based survey of all staff (clinical and non-clinical) was conducted at a hospital undergoing major organizational change in Sydney, Australia. The survey included items regarding teamwork culture, communication (feeling informed, communication channels), change readiness (appropriateness, change efficacy), and burnout. With a sample size of 153 (62% clinical staff), regression and path analyses were used to examine relationships between variables.

**Results:**

The total effects between teamwork culture and burnout was significant [β (Total) = −0.37, *p* < 0.001) and explained through a serial mediation. This relationship was found to be mediated by three factors (feeling informed, appropriateness of change and change efficacy) in a full mediation. Further, change readiness (appropriateness of change and change efficacy) mediated the relationship between feeling informed and burnout. The most useful channels of change communication included face-to-face informal communication, emails, and a newsletter specifically about the change.

**Conclusion:**

Overall, the results supported the predicted hypotheses and were consistent with past research. In the context of large hospital change, staff with a positive teamwork culture who feel informed are more likely to feel change-ready, heightening the chances of successful organizational change and potentially reducing staff burnout. Understanding the pathways on how culture and communication related to burnout during organizational change provides an explanatory pathway that can be used to heighten the chances of a smooth change transition with minimal disruption to staff and patient care.

## 1. Introduction

A supportive organizational culture is considered a key attribute in enhancing the likelihood of success in a program of organizational change (1–3). Organizational culture is defined in different ways, but for our purposes refers to the shared values, thinking, and behaviors of people in workplaces and organizations (4). This differs from organizational climate, which is defined as the shared understanding of policies, practices, and procedures staff experience and expected behaviors (5).

A supportive organizational culture has been described as a work environment that is: trusting and collaborative; prioritizes safety and teamwork; management is supportive and encouraging; and involves employees in decision making (6). In the case of hospital organizational change, a supportive organizational culture may include ensuring that staff in departments across the hospital feel valued, included in, and informed by management about the changes occurring in the workplace. A notable challenge with improving organizational culture in order to heighten the chances of successful organizational change is that culture is not easily changed – and when it can be altered, it usually takes considerable time and resources (7). A successful organizational change can be defined as an initiative having long-term sustainability, and with minimal disruption to the quality and safety of patient care (8). Given that culture is a known predictor of successful organizational change in healthcare (9) it is important to identify factors that can practically influence culture, to ultimately contribute to successful long-term organizational change.

Previous research has emphasized the importance of teamwork as a key indicator of a supportive organizational culture and as a potential factor contributing to the success of organizational change (10). Fostering a culture of teamwork among hospital staff with shared beliefs of collaboration and cooperation will in turn affect their levels of engagement and participation in collective decision making during a change initiative (11). Conversely, lower levels of teamwork and a stressful work environment have been proposed as antecedents for lower engagement and ability to cope with change; ultimately leading to higher levels of burnout and absenteeism among hospital healthcare workers (11, 12).

Another potential factor that may contribute to successful organizational change is related to communication and how informed staff feel regarding the change initiative. Change management communication is viewed as a crucial element for the sharing of change information to raise awareness and increase support for staff during organizational change. Indeed, past research highlights the importance of communication for positive organizational culture and change (13). Effective communication can allay staff fears and uncertainty regarding the change and can foster confidence in their ability to cope with the change (14). Makay et al. (15) identified that timely and effective communication was positively related to staff feeling ready for change, also known in the literature as change readiness. Change readiness has been proposed as “the cognitive precursor to the behaviors of either resistance to, or support for, a change effort” (pp. 681–82) (15). Recent literature has also identified the psychological impact of change attitudes on staff-wellbeing, with staff who felt “not ready” and uninformed expressing fatigue and burnout (8, 16) as a result of the change.

Change readiness refers to the extent to which employees feel prepared for an upcoming organizational change, i.e., feeling the change is appropriate for the organization and that employees are ready to take on the change initiative (17). At an individual psychological level, change readiness in hospitals consists of two key components: (1) appropriateness (healthcare workers perceive that the change is appropriate) and (2) self-efficacy (healthcare workers perceive that they possess the skills and competencies to successfully implement the proposed change) (8). However, various psychological theories (e.g., social information processing models) remind us that the creation of change readiness extends beyond individual cognitions since it involves social phenomena as well; i.e., an individual's readiness for change is also shaped by the readiness of others, and in particular the team members with whom they work most closely. Indeed, there is a growing body of research examining the role of employees' perceptions of broader contextual variables, including organizational culture, in fostering readiness for change. Jones et al. (18) identified that organizational cultures fostering high levels of teamwork were more ready for change, which in turn, predicted post change implementation success (18). Jones et al. (18) further suggested that such teams fostered cohesion and morale through open communication and participative decision making, indicating potential explanatory pathways through which organizational culture positively shapes organizational change.

In order for staff to feel informed and ready for organizational change it is important that change is communicated using appropriate channels (19). According to past research, the most commonly used and preferred channels of change communication are less formalized, face-to-face mediums (19, 20) including small informal discussions (19) staff meetings, and discussions in focus groups or teams (20). Similarly, in healthcare, emphasis has been placed on the desirability of face-to-face meetings, with a need to target clinical leads, key decision-makers and professional teams covering all individuals and groups across a hospital organization (21). Face-to-face meetings provide the opportunity to solicit suggestions, and for healthcare staff to share their perspectives, tender their views and seek clarifications (22). Further, using multiple channels for change communication is useful, broadly (23) and in healthcare specifically (22) to ensure change information reaches as many staff as possible. However, there is an apparent dearth of the literature examining useful channels of change communication in hospital organizational change—i.e., what channels are most useful to communicate organizational change to hospital staff?

The purpose of this paper was to test an explanatory path model for how teamwork culture influences staff attitudes in feeling informed and ready for change, and ultimately leading to reduced staff burnout. The model was developed from survey responses from both clinical and non-clinical staff, at a time that organizational change was occurring in real-time as their hospital underwent redevelopment. A secondary objective was to examine different types of change communication, to identify the channels that were perceived as useful for the communication of changes during this period of large organizational change (i.e., hospital redevelopment).

Based on previous literature, we hypothesized that:

H1. Teamwork culture will have a significant direct positive relationship with feeling informed and change readiness.
H2. Feeling informed and change readiness will have a significant direct negative relationship with staff burnout.
H3. Feeling informed will have a significant, but indirect impact on burnout during organizational change, explained through the mediational role of change readiness.
H4. Teamwork culture will have a significant, but indirect impact on burnout during organizational change, explained through the mediational role of feeling informed and change readiness.

**Figure 1:**
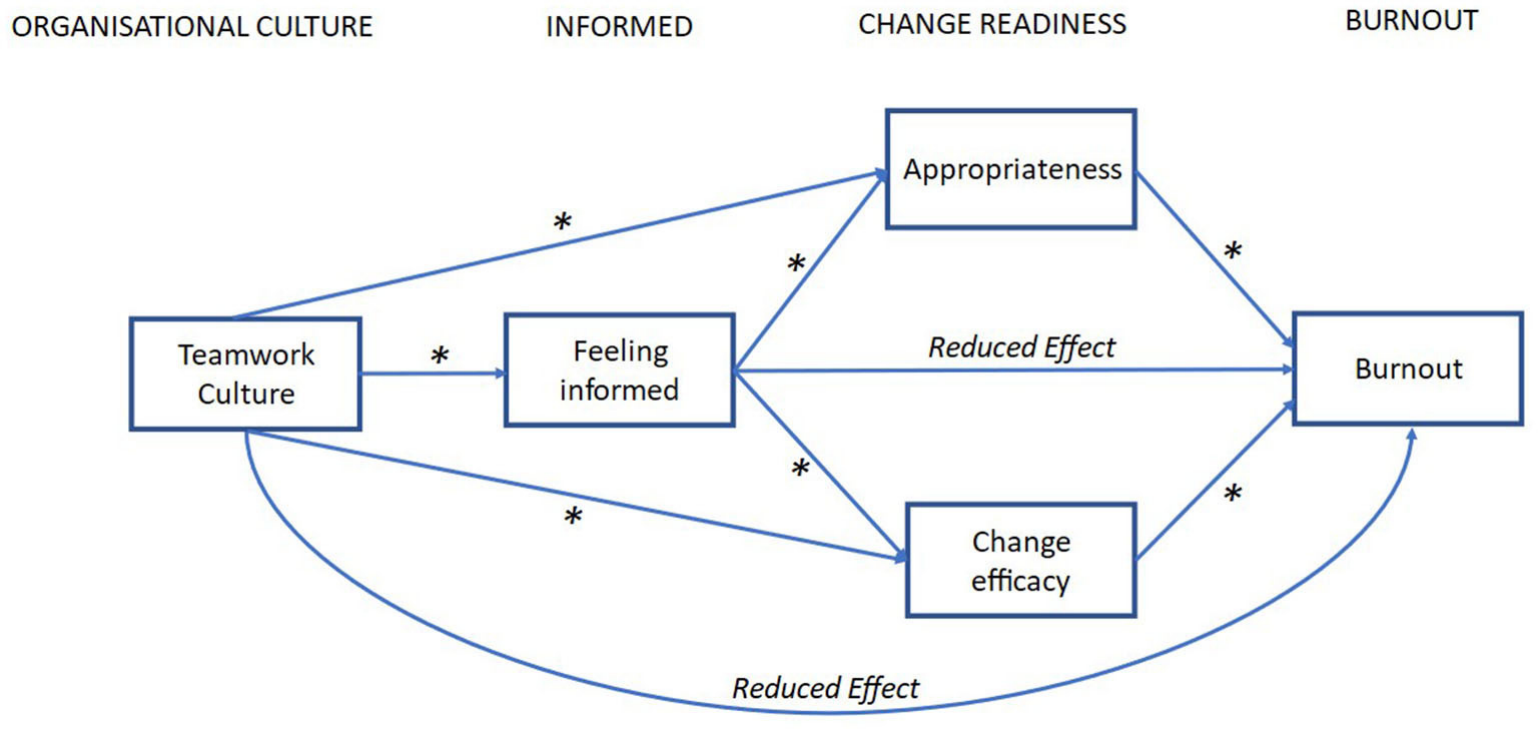
Hypothesized explanatory path model for the influence of teamwork culture on burnout and the role of feeling informed and change readiness during organizational change. The model includes nine direct pathways with two serial mediations to two parallel mediators (change appropriateness and change efficacy); * = hypothesized significant effect.

Figure 1 displays the hypothesized serial mediation model.

## 2. Methods

This study employed a cross-sectional online and paper-based survey of hospital staff from a publicly funded hospital in metropolitan Sydney.

### 2.1. Participants and setting

Participants were staff (clinical and non-clinical) at a hospital in Sydney, Australia. The hospital was undergoing a multimillion-dollar development including the opening of a new hospital building. More detail on the study setting and the change are reported elsewhere (16, 24). Data collection was conducted before the new hospital building opened while staff were undergoing the orchestrated organizational change. Consent was obtained from all participants (written for those who completed the paper based survey and online for those who completed the online survey). Participants understood that their participation was voluntary, confidential and non-identifiable. Participants were assured that they could withdraw from the study at any time without consequences. No reminders were sent to complete the online survey, and unfinished surveys were not included in the data analysis. The study was approved by the relevant Ethics Committee in Sydney, New South Wales, Australia (reference no: 18/233).

### 2.2. Survey

The survey was distributed in both online and paper-based forms in July to August 2019. Online surveys were distributed via email to staff from managers and an online all-staff bulletin. Participants were invited to participate by clicking on a link that led to the survey, which was hosted by Qualtrics (25). In case staff preferred filling in a paper-based survey, these were distributed to hospital staff (clinical and non-clinical) by ward managers and departmental directors.

#### 2.2.1. Teamwork culture

To assess teamwork culture the six-item teamwork climate scale from the widely used Safety Attitudes Questionnaire (SAQ) (26) was adopted. Questions were measured on a five-point Likert-type scale (1 = strongly disagree to 5 = strongly agree). In the present study, we found high internal consistency reliability for the teamwork climate scale (Cronbach's α = 0.81), similar to that reported by Sexton et al. (26) (Raykov's ñ = 0.90) (26).

#### 2.2.2. Change readiness

The validated Hospital Readiness Questionnaire (HRQ) (8) was used to assess change readiness. Two subscales were included: appropriateness (four items) and change efficacy (four items). Items were rated on a seven-point Likert scale (1 = strongly disagree to 7 = strongly agree), with higher scores indicating a greater degree of change readiness. In this study, we found acceptable internal consistency reliabilities for the two HRQ subscales for appropriateness (Cronbach's α = 0.85) and change efficacy (Cronbach's α = 0.74).

#### 2.2.3. Burnout

Burnout was measured using a 10-item version of the Maslach Burnout Inventory (MBI) (27, 28). Due to the inappropriateness of the third subscale, personal accomplishment, for use in healthcare settings (28, 29) only two subscales of burnout—emotional exhaustion (five items) and depersonalization (five items)—were used. Items were measured on a seven-point Likert scale (1 = strongly disagree to 7 = strongly agree). In the present study, the internal consistency coefficients for emotional exhaustion (Cronbach's α = 0.92) and depersonalization (Cronbach's α = 0.86) were both very good.

#### 2.2.4. Feeling informed and channels of change communication

Purpose-designed items were developed to assess how informed staff felt, and what channels of change communication they perceived to be most useful. “Feeling informed” was measured on a four-point Likert scale (1 = very informed, 4 = very uninformed). When asked about useful channels of change communication the following options were provided: chatting with other staff (i.e., face-to-face informal communication), emails, formal presentation, line manager, meeting, newsletter specifically about the change, signs around the hospital, social media, website. These items were created by the research team in collaboration with key stakeholders at the hospital. Specifically, the options for channels of change communication were pre-determined by knowledgeable stakeholders at the hospital. These items were piloted with an expert panel (*n* = 10; researchers with clinical backgrounds and hospital staff not involved as participants in the study) to ensure that the items were applicable and were modified where necessary to improve clarity.

### 2.3. Data transformations and analysis

Some items were reversed coded so that higher item-response scores indicated a greater extent of change readiness and positive organizational culture. While originally measured on a four-point Likert scale, “Feeling informed” was dichotomized for more ready analysis (0 = uninformed, 1 = informed).

Hypotheses were assessed using path analysis to examine the direct and indirect relationships between teamwork culture and burnout, and the mediational role of change attitudes (feeling informed, appropriateness and change efficacy) during organizational change. Analyses were performed using the PROCESS procedure V3.5 (30) in SPSS version 27 (31). From PROCESS, Model 81 was used for the path model. To manage bias, a non-parametric bootstrapping analysis was used to test the null hypothesis for the mediations. Indirect pathways were found to be significant if the 95% bias-corrected confidence intervals for the indirect effects does not cross zero. The model was adjusted for age, sex, and the number of years worked at the hospital. To assess how much of an effect was mediated through the indirect pathway we calculated the mediation proportion, defined as the proportion from the indirect effect (the mediator) on the total effects, that is, the indirect effect divided by the total effect (32). To assess for differences between the parallel pathways, a contrast of the indirect effects was tested (30). Usefulness of communication channels were examined using descriptive and logistic regression analysis in SPSS version 27 (31).

## 3. Results

### 3.1. Descriptive statistics

Two-hundred and eleven surveys were received; only surveys with no missing data for the variables to be used in the path analysis (teamwork culture, change readiness and burnout) as PROCESS requires complete data for analysis, resulting in 153 usable responses (73% effective response rate). Table 1 summarizes demographic and work characteristics of respondents and Table 2 presents the means, standard deviations, skewness, and kurtosis values, and intercorrelations for all variables included in the path analysis. Skewness and kurtosis values were within acceptable ranges of normality. All bivariate correlations were statistically significant and in the hypothesized direction.

**Table 1:** Demographic and work characteristics of respondents (*N* = 153).

		***n***	**%**
Gender	Male	41	27.0
	Female	109	71.7
	Other	2	1.3
Age	18–24 years	8	5.2
	25–34 years	36	23.5
	35–44 years	33	21.6
	45–54 years	44	28.8
	55–64 years	26	17.0
	65+ years	6	3.9
Role	Clinical	93	61.6
	Non-clinical	38	24.8
	Both	20	13.2
Profession	Administration/clerical	20	13.1
	Allied health professional	12	7.8
	Management	17	11.1
	Medical officer/consultant	26	17.0
	Registered nurse/midwife/enrolled nurse	60	39.2
	Other (e.g., cleaning, porter, security, chaplain)	22	14.4
Experience at hospital	<1 year	15	10.1
	1–3 years	37	24.8
	4–6 years	35	23.5
	7+ years	62	41.6

Responses may not equal 153 due to missing data for demographic variables.

**Table 2:** Descriptive statistics and correlations for study variables.

		**M**	**SD**	**SK**	**KU**	**1**	**2**	**3**	**4**	**5**
Culture	1. Teamwork culture	21.3	6.3	0.2	−0.5	–	0.26^*^	0.48^**^	0.38^**^	−0.41^**^
	2. Informed	–	–	–	–		–	0.31^**^	0.33^**^	−0.15
Change readiness	3. Appropriateness	15.8	4.0	−0.8	0.5			–	0.43^**^	−0.48^**^
	4. Change efficacy	17.4	4.8	0.0	0.2				–	−0.49**
	5. Burnout	39.2	15.2	0.1	−0.7					–

* P < 0.05,

** P < 0.001.

### 3.2. Path analysis

**Figure 2:**
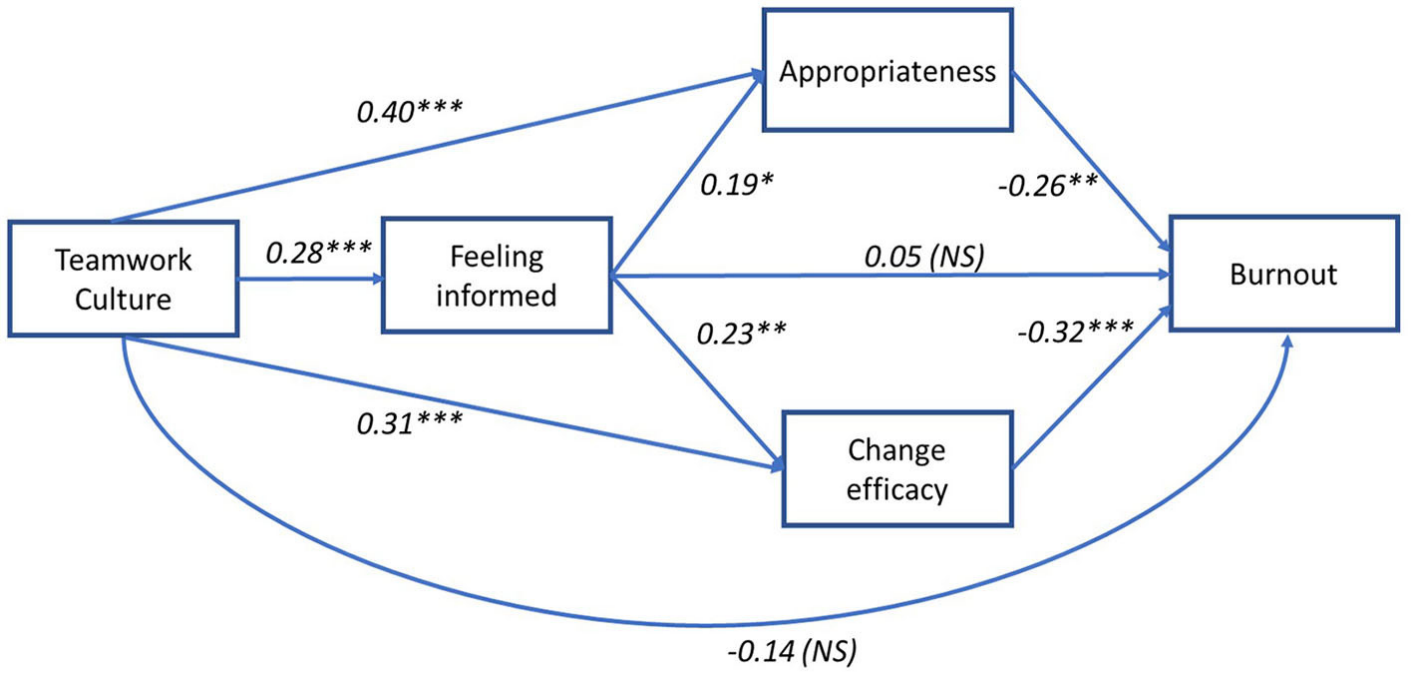
Explanatory path model for the influence of teamwork culture on burnout and the role of feeling informed and change readiness during organizational change with regression coefficients (B). ^**^P < 0.00I, NS, not significant.

Path analysis was used to test an explanatory path model of the study variables (see Figure 1). For the direct pathways, as predicted, the results showed significant positive associations between teamwork culture and the feeling informed and change readiness (appropriateness, change efficacy) mediator variables. There were also significant positive associations between feeling informed and change readiness pathways. Additionally, there were significant negative associations between change readiness variables (appropriateness, change efficacy) and burnout. Further, the direct relationship between feeling informed with burnout, and between teamwork culture with burnout were not significant (see Figure 2). As predicted, four out of five indirect pathways were significant (see Table 3 for details). Results for the model showed a significant total effect between teamwork culture and burnout [β (Total) = −0.37, SE = 0.18, *p* < 0.001), however, the direct effect was not significant [β (Direct) = −0.14, SE = 0.19, *p* = 0.08], indicating that a full mediation has occurred. The three change attitude mediators, feeling informed, change appropriateness and change efficacy fully mediated the relationship between teamwork culture and burnout indicating that the relationship can be explained through the serial and parallel indirect pathways. To examine whether the contributions of the two change readiness variables were different in the parallel pathways, we tested for differences between the two parallel indirect pathways (TW > APP > BO and TW > CE > BO) through pairwise contrasts. We found no significant difference between these two paths [β (contrast) = −0.005, SE = 0.06, 95% CI (−0.118, 0.118)]. We also examined if the two serial indirect pathways (TW > INF > APP > BO and TW > INF > CE > BO) contributed differently through pairwise contrasts and found no significant difference between these two paths [β (contrast) = 0.007, SE = 0.014, 95% CI (−0.019, 0.038)].

**Table 3:** Indirect effects for the indirect pathways between teamwork (TW) and burnout (BO).

**Mediation pathway**	**Standardized indirect effect**	**SE**	**95% CI**	**Mediation proportion**
TW > INF > BO	0.013	0.023	−0.033, 0.062	0.037
TW > APP > BO	−0.104	0.041	−0.188, −0.031	0.283
TW > CE > BO	−0.099	0.040	−0.187, −0.033	0.270
TW > INF > APP > BO	−0.014	0.009	−0.035, −0.001	0.037
TW > INF > CE > BO	−0.021	0.012	−0.049, −0.003	0.056

APP, appropriateness; BO, Burnout; CE, change efficacy; INF, informed; TW, teamwork culture.

### 3.3. Channels of change communication

**Figure 3:**
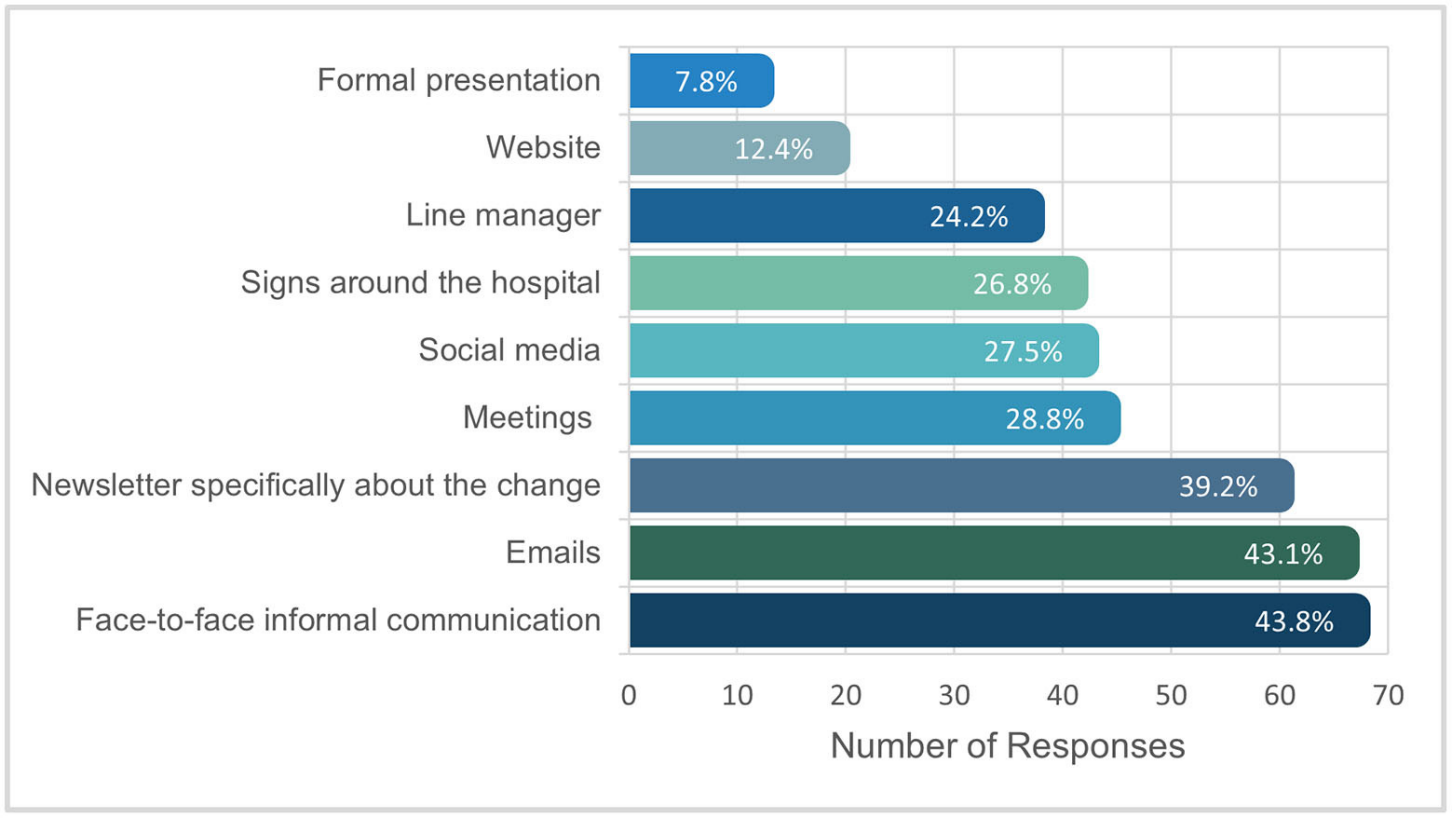
Usefulness of channels of change communication.

Most staff reported that they felt somewhat or very informed (*n* = 96; 64.0%) regarding the hospital organizational change. The most commonly reported useful channels of informing staff about the change were: face-to-face informal communication (*n* = 67, 43.8%), emails (*n* = 66, 43.1%), and a newsletter specifically about the change (*n* = 60; 39.2%). Figure 3 shows the channels of change communication ranked as most useful. Further, most participants indicated that multiple channels were useful (*n* = 101; 66.0%), with less than a third of the sample reporting only one channel as useful (*n* = 47, 30.7%).

Logistic regression analyses were conducted to examine if useful channels of change communication differed across demographic characteristics. First, we examined the likelihood of participants reporting “face-to-face informal communication” as a useful channel of information based on gender, age, role, profession and experience at hospital. The model was statistically significant, χ^2^(18) = 30.05, *p* = 0.037, explained 24.8% (Nagelkerke R2) of the variance and correctly classified 69.4% of cases. Staff that had worked at the hospital < 1 year were 7.26 times more likely to report face-to-face informal communication as a useful means of change communication compared to staff that had worked at the hospital for seven or more years. No other variables were associated with the likelihood of nominating ‘face-to-face informal communication' as a useful channel of change information. Further, the likelihood of hospital staff reporting emails as a useful channel of change information did not significantly differ based on gender, age, role, profession and experience at hospital, χ^2^(18) = 18.57, *p* = 0.419. Lastly, we found that the likelihood of hospital staff reporting the change specific newsletter as a useful channel of change information significantly differed based on gender, age, role, profession and experience at hospital, χ^2^(18) = 33.36, *p* = 0.015, explained 27.5% of the model and correctly classified 71.4% of cases. Allied health professionals were 5.75 times more likely to report the newsletter as a useful channel of change communication compared to nursing staff. Further, staff aged over 65 years were 0.07 and 0.06 times more likely to find the newsletter useful compared to staff aged 25–34 and 35–44 years, respectively.

## 4. Discussion

The aim of the paper was to test an explanatory path model for how teamwork culture influences staff attitudes in feeling informed and ready for change, and ultimately leading to reduced staff burnout. It also identified perceived useful channels of change communication prior to a large hospital organizational change. Overall, the results supported the predicted hypotheses and were consistent with past research.

The explanatory model found positive and significant relationships between teamwork culture and change management communication variables; feeling informed, change appropriateness and change efficacy. This finding supports the role of a positive teamwork culture leading to better change communication. Effective teamwork and communication in healthcare settings have previously been found to play a crucial role in the delivery of safe and high quality care, through focus on a collaborative culture (33). Collaboration, a central tenet of a positive teamwork culture, leads to not only efficient processes but also improved communication (34). Additionally, Simoes and Esposito (35) found that communication needs to be “dialogic” for there to be a reduction in resistance to change, further demonstrating the importance of collaboration.

The model also found that the influence of teamwork culture on burnout was indirectly mediated by both feeling informed and change readiness. Poorer teamwork culture was directly associated with burnout during organizational change, however, focus on effective change communication could ultimately mitigate this relationship and contribute to reduction of burnout. The relationship between feeling informed and burnout was also mediated by change readiness. This shows that it is more than just feeling informed that contributes to positive organizational culture in hospital organizational change. Staff also need to perceive the change as appropriate and that they are capable of dealing with the change for there to be a positive impact on organizational culture, and ultimately heighten the chances of a smooth change transition with minimal disruption to patient care. Readiness for change has received much attention in the organizational change literature (35) for its contributary role in successful organizational change (36). This study provides further support for the important role of change readiness in organizational change as a mediator for positive organizational culture in the oftentimes chaotic time of hospital organizational change.

For hospital staff experiencing the early stages of large-scale organizational change, the most useful channels of change communication were face-to-face informal communication, emails, and a change specific newsletter. Face-to-face informal communication was the most commonly reported useful channel of change communication, particularly so for staff that had worked at the hospital < 1 year. This is consistent with theories of communication maintaining that informal communication networks are important during change programs (37). particularly when both the sender and receiver are able to clarify their understanding (38). For new hospital staff, being able to partake in an active, two-way conversation where clarification can be sought is vital to ensure staff feel informed and equipped for the organizational change. Further, this sheds light on another way to ensure hospital staff feel informed in the lead up to organizational change: by leveraging change agents. Change agents, otherwise termed “champions” or “brokers” in the healthcare literature, can be used to transfer information across boundaries (professions, wards, day/night shifts) (39) and are integral in the adoption and diffusion of new phenomena (40–42). Change agents are essential for the success of organizational change because of their collaborative power (i.e., ability to bridge boundaries and pass on information) and advocacy (i.e., spreading a positive message about the change). We also found that most staff (*n* = 101/153) reported multiple channels of change communication as useful (as opposed to only reporting one useful channel), supporting past healthcare literature emphasizing the importance of using multiple channels of change communication for successful organizational change (22). We also found differences between professionals in terms of what channels of change communication were deemed most useful. This reinforces the importance of using diverse and multiple channels of change communication to ensure change-related information reaches as many staff as possible.

### 4.1. Implications

This study highlights that the way in which organizations communicate with their employees during organizational change can have significant effects on organizational culture and the success of change and *vice-versa*. Key principles to ensure hospital staff feel informed and ready for organizational change include using multiple channels of change communication (e.g., encouraging face-to-face informal communication as well as emails between staff and a change specific newsletter) and preparing key people to be change agents with the brief of face-to-face informal communication among staff and making themselves available for discussion about the change. These recommendations can be used by managerial staff working through hospital change.

### 4.2. Strengths and limitations

A methodological strength of this study was the use of path analysis to test the influence of teamwork culture on burnout and the role of feeling informed and change readiness during organizational change. This is also one of the first studies to identify and recommend useful channels of change communication in hospital organizational change. A limitation was that the effectiveness of the channels of change communication was not directly measured. There are a lack of appropriate and rigorous tools that assess effective change communication, (43) therefore, we relied on the self-reports of hospital staff *via* an author-developed survey tool to identify which channels they perceived as most useful. Further, the explanatory between teamwork culture and burnout was performed through a cross-sectional survey; as such, it is based on staff perceptions at one-point in time. While the model explains the mediating effects of change attitudes (feeling informed, appropriateness and change efficacy) during organizational change, it does not take into account other factors that may impact burnout, such as workload. This means that the results need to be interpreted with caution until they have been replicated in follow-up longitudinal research. Another limitation is that the findings may be restricted to the contextual subtleties of the hospital and the specificities of the hospital redevelopment. Finally, given that the survey was advertised *via* email within the hospital bulletin and paper-based surveys were distributed to hospital staff by ward managers and health professional directors, we were unable to calculate a response rate, and we could not determine whether there was a difference between participants and non-participants. Nevertheless, the study was designed to produce nuanced, in-depth data with aspects transferable to other instances of large-scale hospital change. The research is applicable to other hospitals, particularly in Australia's most populous state, New South Wales, where there are approximately 30 large public hospitals that have similar organizational structure in terms of funding, administration and staff skill mix.

## Conclusion

In conclusion, this study highlights the importance of positive teamwork culture, as well as change readiness in heightening the chances of successful organizational change and reducing staff burnout. A key implication from this study is that while building a positive organizational culture typically takes time, managers can seek to reduce burnout by improving change communication and ensuring staff feel informed and ready for the organizational change.

## Data availability statement

The raw data supporting the conclusions of this article will be made available by the authors, without undue reservation.

## Ethics statement

The studies involving human participants were reviewed and approved by the relevant Ethics Committee in Sydney, New South Wales, Australia (reference no: 18/233). The patients/participants provided their written informed consent to participate in this study.

## Author contributions

CP collected the data. LE, YT, and CP analyzed the results and wrote the first draft of the manuscript. KC, JL, JB, and MS provided critical feedback and helped to shape the final manuscript. All authors contributed to the article and approved the submitted version.
